# Optimizing life success through residential immersive life skills programs for youth with disabilities: study protocol of a mixed-methods, prospective, comparative cohort study

**DOI:** 10.1186/s12887-016-0694-7

**Published:** 2016-09-06

**Authors:** Amy C. McPherson, Gillian King, Alanna Rudzik, Shauna Kingsnorth, Jan Willem Gorter, Ontario Independence Program Research (OIPR) team

**Affiliations:** 1 Bloorview Research Institute, Holland Bloorview Kids Rehabilitation Hospital, 150 Kilgour Road, Toronto, ON M4G 1R8 Canada; 2 Dalla Lana School of Public Health and Rehabilitation Sciences Institute, University of Toronto, Toronto, ON Canada; 3 Department of Occupational Science and Occupational Therapy, University of Toronto, Toronto, ON Canada; 4 Department of Pediatrics, CanChild Centre for Childhood Disability Research, McMaster University, Hamilton, ON Canada

**Keywords:** Life skills, Transitions, Disabilities, Youth, Protocol, Adulthood, Qualitative research, Environment, Therapy, Self-determination, Self-efficacy

## Abstract

**Background:**

Young people with disabilities often lag behind their typically developing peers in the achievement of adult roles, which has been attributed to a lack of opportunities to develop critical life skills. Residential Immersive Life Skills (RILS) programs provide situated learning opportunities to develop life skills alongside peers and away from home in real-world settings. Retrospective research suggests that attending RILS programs is a transformative experience that empowers youth, provides parental hope, and increases service provider expertise. However, prospective, comparative research is needed to determine longer term benefits of these programs on youth life trajectories, in addition to exploring the program features and participant experiences that optimize program success. This protocol describes a 5-year, multi-site prospective study examining the effects of RILS programs for youth with disabilities.

**Methods:**

The study involves RILS programs at three sites in Ontario, Canada. Cohorts of treatment and control groups will receive the study protocol over 3 successive years. Thirty English-speaking participants aged 14–21 years with a child-onset disability and the cognitive capacity to engage in goal setting will be recruited every year for 3 years in the following groups: youth attending a RILS program (Group A); a deferred RILS control group of youth (Group B); a control group of youth attending a non-residential life skills program (Group C); and a control group matched on age, diagnoses, and cognitive capacity not receiving any life skills intervention (Group D). All participants will complete measures of self-determination and self-efficacy at four time points. Program opportunities and experiences will also be assessed in-the-moment at the RILS programs. Qualitative interviews pre-program and at 3- and 12-months post-program will be undertaken with a sub-sample of youth and parents to explore their expectations and experiences.

**Discussion:**

This study will address key gaps in the literature pertaining to the long-term impact of RILS programs and the role of immersive environments in shaping youth outcomes and experiences. Our research program aims to uncover transferable processes and essential features by which RILS programs have their effects on attitudes, cognitions, and behaviour.

**Trial registration:**

The trial registration number on clinicaltrials.gov is NCT02753452 (retrospectively registered 26 April 2016).

Trial sponsor: Holland Bloorview Kids Rehabilitation Hospital.

## Background

In Canada, between 3.5 and 7.7 % of children under 19 years of age are estimated to have a chronic health condition that results in disability or activity limitations [1, 2]. Despite having the same life aspirations as their typically developing peers [3, 4], they often lag behind them in achieving adult roles with respect to independent living, personal relationships, education, and employment [5–7]. A combination of physical, cognitive, and social restrictions result in a lack of opportunities and experiences needed by youth with disabilities to acquire the life skills vital for the achievement of adult roles [8]. This protocol outlines a 5-year multi-site prospective cohort study that will examine the effects of residential immersive life skills (RILS) programs for youth with disabilities. The study will contribute to our understanding of how these programs provide youth with the opportunities and experiences needed to attain successful life outcomes. The protocol adheres to the SPIRIT guidelines for reporting of clinical trial protocols (http://www.equator-network.org/reporting-guidelines/spirit-2013-statement-defining-standard-protocol-items-for-clinical-trials/).

Life skills are adaptive, positive behaviours that enable an individual to meet the challenges and demands of everyday life effectively [9]. Two key life skills targeted by life skills programs are self-determination and self-efficacy [10, 11]. Self-determination refers to the ability to make choices and have control over one’s life [12]. A strong sense of self-determination is associated with improved life outcomes among youth with disabilities [13, 14]. Self-efficacy-a central component of self-determination theory-is the feeling of being capable of taking action and coping with challenges [15]. When youth lack opportunities to tackle and succeed in mastering day to day tasks, this can lead to an underdeveloped sense of self-efficacy, which has been associated with poorer psychosocial outcomes [16, 17]. Conversely, mastering developmentally appropriate skills has been found to lead to greater psychological wellness in youth [18]. Young people with disabilities may be prevented from exploring independently or testing their strengths by parents worried for their safety or about the impact failure may have on them [19]. Unfortunately, such restrictions may result in youth having lowered expectations of themselves and performing below their capabilities [20]. Program models that combine the challenge of real-world settings with opportunities for exerting control may therefore be particularly effective for the development of youth self-determination and self-efficacy [21].

Life skills programs have targeted many vulnerable populations to support the development of critical skills during adolescence and young adulthood. They typically include a combination of structured group education sessions, coaching, and peer mentorship [22]. Through teaching concrete tasks such as budgeting and laundry that will equip young people with the skills needed for independent living, the programs aim to develop higher order skills such as self-efficacy and self-determination.

### Residential immersive life skills (RILS) programs

RILS programs are a unique type of life skills program for youth with disabilities and provide opportunities for learning with peers in a university or college residence, for periods between one and three weeks. RILS programs use flexible, multi-faceted approaches to provide experiences in choice-making, facilitated goal-setting, trial and error learning, and risk taking [22]. RILS programs appear to provide opportunities for ‘situated learning’, a powerful educational approach where learning occurs within the environments where the skills will be needed [23], which increases their potential to provide sustainable gains. Expert service providers report intentionally creating a safe, supportive, and engaging environment at RILS programs and tailoring learning strategies to the needs of the youth [24]. Non-intrusive strategies are common, such as allowing youth to ‘fail safely’, thus experiencing the consequences of their decisions in a supported environment. This can result in feelings of empowerment and self-determination [24].

The residential nature of RILS programs offers repeated opportunities to master skills and also allows youth to learn skills as part of their everyday life, termed ‘experiential learning’ [25]. RILS programs also facilitate interactions with peers with disabilities, offering intense learning and social experiences that go beyond skill development [26]. For some participants, this is one of their first opportunities to engage with others with disabilities, which may lead to greater understanding of their identity [27]. RILS programs often result in profound changes in young people and have been described as a ‘turning point’ in their transition to adulthood [28]. Secondary benefits may also arise: Service providers report their own clinical practice to be positively impacted by spending extended time with the youth and witnessing their daily reality, while parents often experience a transformation in their hopes and expectations for their child’s future [28].

A pilot study by our team previously used a range of measures to capture the environmental opportunities, in-the-moment experiences, and intervention strategies used in an established RILS program [29]. Findings demonstrated high program fidelity i.e., it was delivered and experienced as intended. There was also evidence that the RILS program provided a facilitative environment enabling youth to develop autonomy, self-determination, and self-efficacy. This pilot work therefore informed the design of the protocol described here and confirmed the suitability of the selected measures, which will be able to examine program features and experiences that may contribute to youth outcomes over time.

### Research gap

Although the findings to date are promising with regard to the impact of RILS programs, the unique immersive nature of RILS programs, which may enhance or accelerate the development of life skills, has not been examined. Furthermore, research has focused on short-term changes related to self-determination, self-concept, and preparedness for life [22, 30]. There is a lack of research on the sustainability and/or consolidation of skills acquired in the programs, and the emergence of higher-order life skills after additional practice and reflection. Critically, the processes and essential features by which RILS programs have their effects on attitudes, cognitions, and behaviour require further explication, including the role of peer interactions. Finally, research has largely been retrospective in nature (e.g., [24, 27, 28]).

### Objectives

Our objectives are to understand the ways in which RILS programs lead to changes in youth outcomes and to determine the sustainability of these changes. In addition, we will identify the processes and intervention strategies integrated into program delivery, and explore the experiences of participants and their parents before, during, and after the program.

### Aims

Our specific study aims are as follows:

1. To examine the nature of the opportunities provided by RILS programs for particular types of youth experiences, and the extent to which service providers use specific learning strategies to promote the acquisition of core skills, knowledge, and behaviours.
2. To explore the subjective experiences of youth and parents before, during, and after the program.
3. To assess change and/or maintenance in outcomes, namely youth development of higher-order life skills (self-efficacy and self-determination) up to 1 year post-program, when compared to three groups of matched youth.

## Methods/design

### Research design

The study uses a mixed-methods approach to assess the complex opportunities, experiences, and outcomes of RILS programs [31]. This 5-year multi-site prospective study will employ a time series with non-equivalent control group (TSNECG) design, which will control for many threats to validity and allow reasonable attribution of the effects to the RILS program intervention [32]. Specifically, the TSNECG design will allow us to determine whether exposure to RILS programs has an effect on higher-order life skills compared with the three comparison groups, assessed from pre-intervention to immediately post-intervention, and at 3- and 12-month follow-up assessments.

### Settings and intervention

Three core organizations in the province of Ontario, Canada, deliver well-established RILS programs. Program coordinators from these three organizations have collaborated for over 10 years to develop the programs and thus they share common goals and a common philosophy.

The programs run once per year over the summer. Each is delivered by a team of service providers, including occupational and physical therapists, therapeutic recreation specialists, life skills facilitators, nurses, social workers, and personal attendants. All programs take place in a university or college residence for periods of one to three weeks.

Although self-determination theory was not explicitly used in the initial design of the RILS programs, repeated opportunities for youth to make decisions and to practice daily tasks (e.g., budgeting, laundry, directing personal care, cooking, navigation, meal planning) aim to promote the development of self-determination and self-efficacy.

To participate in the programs, youth must have a child-onset disability, the cognitive capacity to set goals, and must not have behavioural issues that would interfere with their ability to participate in a group learning experience. Youth with mild cognitive impairments, as assessed by clinicians, are eligible to participate. The age range for participation in the programs is 14 to 21 years. Past participants in the three RILS programs have had diagnoses of cerebral palsy (50 %), spina bifida (15 %), acquired brain injury (5 %), communication disorders (5 %), as well as other conditions (25 %).

### Research participants

Participants will be enrolled in four groups, with new participants recruited each year for three successive years. For practical and ethical reasons, we cannot randomly assign youth to treatment and control conditions.

Group A will consist of participants drawn from the youth enrolled in the three RILS programs. All youth will be English speaking, as this is the language of instruction of the RILS programs. In addition to meeting the eligibility requirements for program participation, as described above, youth who wish to participate in the research project will be screened separately by the research coordinator for their capacity to provide informed consent to participate in the study. Parents of youth in Group A must be English speaking to participate in interviews.

Group B will consist of youth who apply and are eligible to participate in one of the RILS programs but who are placed on a ‘deferred’ list to attend the program in the following year. This can be for a number of reasons, including numbers of places available and demand, extent of care needs, and youth availability. This group is intended to control for the motivation participants have for taking part in a RILS program.

Group C will consist of youth taking part in a group life skills program that is not residential. Several life skills programs at treatment centres in Ontario run over multiple days/weeks and employ a group format without being residential. This group will allow us to examine the relative effect of the immersive component of the RILS programs.

Group D, the control group, will consist of clients who are not participating in any group life skills program but are registered with one of six Ontario children’s treatment centres (the three core centres offering RILS programs, plus three additional collaborating centres).

Youth in the three comparison groups (Groups B, C, and D) must meet the eligibility criteria for the RILS programs, to ensure comparability with Group A. They will be matched to the RILS group in terms of age, gender, and diagnosis.

### Instruments

#### Quantitative data

We will collect baseline demographic information on all participants, including youth age, disability, mobility, health status, ethnic or cultural background, where the youth is living and with whom, and the size of their home community.

At baseline, and at three follow-up time points, all youth will complete measures of self-determination and self-efficacy using an online survey. The Arc’s Self-Determination Scale (SDS) adolescent version includes four subscales (autonomy, self-realization, empowerment, and self-regulation) and provides an overall score of self-determination [33]. Self-determination percentile scores can be generated based on norms from a population of US special education students (ages 14 to 22 y) with a wide range of disabilities. Internal consistency is high (Cronbach’s alpha = 0.90) and criterion-related validity is adequate (*r* = 0.25 to 0.5) [34]. The youth will complete all of the subscales except the self-regulation subscale, which uses a story-writing format requiring subjective scoring and can be sensitive to geographic, cultural, and socioeconomic differences among participants [33]. The General Self-efficacy Scale (GSE) is a 10-item self-administered tool with a 4-point Likert response scale [35] and has been used in numerous research studies to examine perceived self-efficacy. It is valid and reliable with internal consistencies ranging from 0.75 to 0.91.

In addition, a number of measures will be collected on-site during the RILS programs for Group A participants. Trained observers will complete the 48-item Measure of Environmental Qualities of Activity Settings (MEQAS-48) to capture program opportunities. Validated with life skills program activity settings, the MEQAS is a reliable and valid measure of aesthetic, physical, social, and opportunity-related qualities of leisure activity settings for youth with or without disabilities [36]. Observers use a 7-point scale to rate the extent to which the observed activity setting includes various features. The MEQAS has a sound factor structure and preliminary evidence of internal consistency, inter-rater reliability, and test-retest reliability. Cronbach’s alpha for the scales (Comfortable Place-related Qualities, Pleasant Physical Environment, Opportunities for Choice, Opportunities for Privacy/Relaxation, Opportunities to Interact with Peers, Opportunities for Personal Growth, Opportunities for Physical Activities, Opportunities for Cooperative Group Activity, and Opportunities to Interact with Adults) range from 0.82 to 0.97 [36]. Inter-rater reliability and test-retest reliability were established on a previous version, the MEQAS-32, with inter-rater reliability (ICC) ranging from 0.60 to 0.93 and test-retest reliability ranging from 0.70 to 0.90. The MEQAS-48 is available at https://flintbox.com/public/project/25723/

Trained observers will also complete the Service Providers’ Strategies-Checklist (SPS-C), a 24-item checklist that captures service providers’ actual use of intervention strategies in life skills programs, including teaching/learning techniques, handling/physical interventions, socially-mediated strategies, cognitive strategies, and non-intrusive strategies. Items were constructed based on service providers’ descriptions of their use of strategies in a qualitative study of RILS service providers and an existing checklist of intervention strategies for school-based therapy [37]. Content validity of the SPS-C was established through review by RILS service providers and clinicians on the research team, and it has a high inter-rater agreement (96 %). The SPS-C will allow us to characterise and compare the strategies used by service providers across the RILS programs. The SPS-C is available at http://flintbox.com/public/project/27313/

To capture in-the-moment experiences during the program, youth will complete the Self-Reported Experiences of Activity Settings (SEAS) measure [38] following selected program activities. The SEAS is a 22-item, youth-completed measure of situation-specific experiences, assessing Personal Growth, Psychological Engagement, Social Belonging, Meaningful Interactions, and Choice & Control. Internal consistency of the SEAS is good to excellent (Cronbach’s alpha from 0.71 to 0.88), with moderate test–retest reliability (mean scale intra-class correlation coefficient = 0.68) [38]. The SEAS is available at https://flintbox.com/public/project/25724/

The schedule of data collection for the study is shown below, in Table 1. All of the measures have been piloted in a RILS program [29]. This pilot work indicated that RILS program features and youth experiences can be reliably and meaningfully assessed *while they are occurring,* and the convergence between the measures demonstrated that youth experienced the program as service providers intended. The measures employed in this current study therefore have demonstrated high feasibility [29].

**Table 1:** Schedule of research activities by group

Research Activities	Baseline	During Life Skills Programs	Follow-up		
			1 week post-program	3 months post-program	12 months post-program
Enrollment:					
Eligibility Screen	A, B, C, D				
Informed Consent	A, B, C, D				
Demographic Form	A, B, C, D				
Intervention:					
Life Skills Programming		A, C			
Life Skills Program Assessments:					
Service Providers’ Strategies-Checklist (SPS-C)		A			
Measure of Environmental Qualities of Activity Settings (MEQAS)		A			
Participant Assessments:					
Self-Reported Experiences of Activity Settings (SEAS)		A			
Arcs’s Self-Determination Scale	A, B, C, D		A, B, C, D	A, B, C, D	A, B, C, D
General Self-Efficacy Scale (GSE)	A, B, C, D		A, B, C, D	A, B, C, D	A, B, C, D
Qualitative Interview	A, C			A, C	A, C

**Group A**: Participants drawn from the youth enrolled in Residential Immersive Life Skills programs*.* **Group B**: Participants drawn from the youth deferred from Residential Immersive Life Skills programs*.* **Group C**: Participants drawn from youth enrolled in non-residential Life Skills programs*.* **Group D**: Participants not participating in any group life skills program

#### Statistical power

We will recruit 30 youth in each of the four study groups in each of the 3 years of recruitment. This will result in a total of 90 youth participants per group. We have allowed for a liberal attrition rate of one-third, which will yield a minimum enrolment of 60 youth per group, sufficient for all analyses. A sample of 50 participants in each group will provide adequate power to detect moderate effect sizes (d = 0.50) on the primary outcome of self-determination, based on the following assumptions: power = 0.80, β = 0.20, α = 0.05 (one-tailed) [39]. Therefore, our projected sample of 60 youth will be sufficient to show any statistically significant changes in outcomes.

#### Qualitative data: young people’s experiences of life skills programs

Fifteen randomly selected participants in Group A (RILS group) and Group C (non-residential Life Skills group) will participate in a series of semi-structured interviews before their program starts, with follow up interviews held 3 and 12 months after their program finishes. Thirty (RILS = 15, non-RILS = 15) interviews at each time point will provide adequate data (*n* = 90 total interviews) for qualitative themes to be generated and compared between time points and between groups. Theme saturation will be assessed throughout the interviewing and analysis process [40].

The interviews will last between 30 and 60 min and be conducted with the participants in their homes or by telephone, depending upon location and preference. The interviews will follow a guide developed and previously tested during pilot research with youth participants from the RILS program at the host institution [41], as well as input from knowledge users on the study team (i.e., program coordinators). The interviews will explore the following: *Pre-program*: youths’ expectations and motivations for attending; *Three months post-program:* activities and experiences they engaged in during the program, and their initial post-program experiences and reflections; *12 months post-program*: their experiences and continued reflection having had the opportunity to use the skills and learnings. Youth will be asked to reflect upon the opportunities and experiences provided during the program, and after the program finished. They will also be asked about the importance of informal peer interactions. All interviews will be digitally audio recorded and professionally transcribed verbatim.

We will also interview a parent/guardian (hereafter ‘parent’) of ten Group A (RILS program) youth participants to obtain another perspective on the program and to ascertain parent expectations and perceptions of program opportunities, experiences, and outcomes. Parents will be interviewed at the same points as the RILS youth-before the program commences, 3 months post-program, and 12 months post-program.

### Data analysis

#### Quantitative analysis

Data from the online instruments (SDS and GSE) will be downloaded into an Excel spreadsheet. Data collection for the SPS-C, MEQAS, and SEAS will use hard copies of the instruments, with data entered into the Excel spreadsheet for data management. The Excel file will then be imported into SPSS for analysis. A portion of the data will be double entered to check for accuracy. The data from the two outcome measures (SDS and GSE) will be analysed descriptively to report means and standard deviations, pre, post, and at both follow-ups (3 and 12 months). The amount of change in the outcomes will be determined across the data collection time points. Baseline (pre-program) scores of all groups will be compared using t-tests to determine whether there are significant differences between groups on the dependent variables. Attrition rates for the groups will be calculated, to determine whether differential attrition is an issue. The RILS group (Group A) will be tested against one comparison group (Groups B, C or D) in each analysis. The primary analysis will therefore be a series of two between (study groups) and three within (longitudinal measurement points) repeated measures analyses of covariance (ANCOVAs), controlling for baseline scores, with the outcome measures as the dependent variables. Effect sizes will be calculated based on difference in pre- to post-test means, using Cohen’s D, with effect sizes between 0.30 and 0.50 considered medium and those >0.60 considered large [42]. We will use multilevel modeling to assess the proportion of variance in our outcome measures associated with the three program sites.

We will describe RILS program features by calculating means and standard deviations of the MEQAS scores and examining patterns of strategy use in the SPS-C data. The SEAS data for individual youth will be collapsed within each activity setting to provide an aggregate activity profile. We will examine convergence and correspondence between the SEAS and the SPS-C and/or MEQAS. Our previous pilot work demonstrated convergence between scores on the MEQAS (observable program features) and SPS-C (observable service provider strategies) [29]. Scores on the SEAS were also further enhanced by youth-reported experiences captured during brief interviews. These initial indicators of conceptual congruence will be further explored during this study.

#### Qualitative analysis

Short debrief interviews from during the program, contemporaneous field notes, and verbatim transcripts of the in-depth interviews will form the data set for rigorous qualitative analysis, using NVivo for data management. This will provide an in-depth understanding of both parent and youth views of opportunities, experiences, and outcomes related to RILS versus non-residential life skills programs. The multidisciplinary team will play a key role in the synthesis and interpretation of the data [43]. Team members will read through each anonymised transcript several times and note emerging themes and patterns. Initial deductive codes related to opportunities, experiences, and outcomes will be identified. Inductive coding will then be used to identify other concepts that relate to the research questions. A list of themes will be generated then reviewed by multiple team members, refining and clarifying themes and categories iteratively. Dissenting views and ‘negative cases’ will be included. Once a final coding scheme has emerged, all documents will be re-coded by a single researcher. We will examine changes and correspondence of perceptions in the sets of parent and youth pre/post transcripts. An audit trail will be maintained to document methodological decisions and contextual notes [44]. Negative case analysis, debriefing sessions, and the involvement of expert team members who have familiarity with the participating programs will all enhance credibility, a key tenet of trustworthiness in qualitative research [45]. The triangulation of different data sources and identification of the team’s assumptions and beliefs will promote confirmability [46]. Although qualitative work aims to be conceptually generalizable rather than empirically generalizable [47], in-depth contextual information will be provided when reporting the qualitative study findings to enhance transferability [48].

#### Mixed-methods analysis

We will integrate quantitative and qualitative data at the level of interpretation. Qualitative and quantitative methodologies offer different strengths [49]. We will triangulate between complementary methods to increase the breadth and depth of our understanding of RILS programs [50]. We will examine qualitative themes addressing our overall objective of elucidating program opportunities, experiences, and outcomes. Profiles of MEQAS and SPS-C data will be examined in conjunction with qualitative themes concerning program opportunities; SEAS data will be examined in conjunction with qualitative themes concerning experiences; and links between these data and outcomes will be identified.

## Discussion

Our program of research and the longitudinal study that we have described in this protocol address key gaps in the literature relating to the long-term impact of RILS programs and the role of immersive environments (i.e., provided opportunities and youth experiences) in shaping youth outcomes. There is little existing research identifying the ‘key ingredients’ of RILS programs, programs that anecdotal evidence describes as being powerful ‘turning points’ in the lives of young people, their parents, and service providers [24, 27]. To our knowledge, this will be the first study to prospectively follow young people to examine changes in higher order life skills over time, comparing outcomes among youth in multiple control groups.

The research has significant implications for understanding how to optimally design and deliver RILS programs for youth with disabilities, through the exploration of program features and opportunities. Findings will make a unique contribution in the following ways: first, this theoretically grounded study will provide important new information about the fundamental processes by which RILS programs influence short-term and longer-term changes in youth. It is important to understand these processes because they are then potentially transportable to guide the development of effective programs for youth with disabilities, but also for other at-risk youth. Second, the research will explore the specific nature of the learning opportunities provided by these programs and how they impact youth experiences. Our understanding of the strategies used by service providers will also be extended from our earlier work [24]. Pilot work indicates that RILS programs accelerate the development of life skills, which will be further explored in this study. One of the strengths of the study is the use of reliable and valid measures of observable program features and intervention strategies. Our findings will support evidence-informed service not only in Ontario where the study RILS programs are based, but nationally and internationally, leading to improved life outcomes for youth with disabilities.

### Limitations

We aim to recruit youth who are dealing with a range of complex health conditions. As in any longitudinal study, there is the potential that some participants will withdraw, due to changing desires to participate, or be lost to follow-up. However, we have put in place incentives for participants to remain involved through the full 1-year period. The youth in the RILS group are involved in the highest number of research tasks; however, the most time-intensive period of research involvement occurs during the RILS programs, which is near the beginning of the 1-year period. This may help to minimise the number of youth who decide to withdraw. Baseline data will be retained for participants who withdraw, so that comparisons may be made between them and the youth who choose to remain involved.

### Ethics

Ethical approval has been obtained (see below for details). All youth, parents and service providers who are involved in the study will provide written informed consent to a research team member prior to the completion of any study activities. Identifying information for potential participants will be stored on an encrypted server, in a password protected folder. If the potential participant opts not to participate in the study, his/her identifying information will be deleted immediately. Identifying information for those who agree to participate in the study will be stored on the same encrypted server, in a password-protected folder that requires authorisation as a member of the research team. This information will be retained for the life of the study and will be used to provide participants with results of the study upon its completion.

### Knowledge mobilization

We will be taking an integrated knowledge mobilization approach, whereby many of the ultimate knowledge users are members of the research team [51]. We will also be employing a combination of strategies in order to make this research accessible to youth and families, service providers, researchers, educators, policy makers, and the public. We will use a knowledge translation planning template [52] to guide our activities with the aim of promoting awareness of the critical importance of life skills development, broadening understanding of the needs of youth with disabilities, influencing future residential immersive program design and development, and building research skill capacity and partnerships. A key communication tool is a devoted project website (OIPR.ca) and Twitter™ handle (@OIPR_ca) designed to house and push study outputs (i.e., lay summaries, infographics, slide decks) to a broader audience. Google analytics will be examined to assess uptake by end-users.

## Abbreviations

RILS: Residential immersive life skills

## References

[1] Canadian Institutes of Child Health (2010). The health of Canada’s children: A CICH profile. Children and youth with disabilities.

[2] McDougall J, King G, De Wit D, Miller L, Hong S, Offord D, Meyer K (2004). Chronic physical health conditions and disability among Canadian school-aged children: A national profile. Disabil Rehabil.

[3] Cussen A, Howie L, Imms C (2012). Looking to the future: Adolescents with cerebral palsy talk about their aspirations-a narrative study. Disabil Rehabil.

[4] King G, Cathers T, Miller Polgar J, MacKinnon E, Havens L (2000). Success in life for older adolescents with cerebral palsy. Qual Health Res.

[5] Gorter J, Stewart D, Woodbury-Smith M, King G, Wright M, Nguyen T, Swinton M (2014). Pathways toward positive psychosocial outcomes and mental health for youth with disabilities: A knowledge synthesis of developmental trajectories. Can J Commun Ment Health.

[6] King G, Baldwin P, Currie M, Evans J (2005). Planning successful transitions from school to adult roles for youth with disabilities. Child Health Care.

[7] Tuffrey C (2013). Adolescents with physical disability: Seeing the individual in context. Arch Dis Child.

[8] Stewart D, Law M, Rosenbaum P, Willms D (2001). A qualitative study of the transition to adulthood for youth with physical disabilities. Phys Occup Ther Pediatr.

[9] World Health Organization, Skills-based health education including life-skills: An important component of a child-friendly/health-promoting school. Geneva: World Health Organization; 2003.

[10] Ankeny EM, Lehmann JP (2011). Journey toward self-determination: Voices of students with disabilities who participated in a secondary transition program on a community college campus. Remedial Spec Educ.

[11] Carter E, Lane K, Pierson M, Glaeser B (2006). Self-determination skills and opportunities of transition-age youth with emotional disturbance and learning disabilities. Except Child.

[12] Burstein K, Bryan T, Chao P-C (2005). Promoting self-determination skills among youth with special health needs using participatory action research. J Dev Phys Disabil.

[13] Wehmeyer M, Palmer S (2003). Adult outcomes for students with cognitive disabilities three-years after high school: The impact of self-determination. Educ Train Dev Disabil.

[14] McDougall J, Evans J, Baldwin P (2010). The importance of self-determination to perceived quality of life for youth and young adults with chronic conditions and disabilities. Remedial Spec Educ.

[15] Bandura A, Ramachaudran V (1994). Self-efficacy. Encyclopedia of human behavior.

[16] Cramm J, Strating M, Roebroeck ME, Nieboer A (2013). The importance of general self-efficacy for the quality of life of adolescents with chronic conditions. Soc Indic Res.

[17] Shapiro D, Martin J (2010). Athletic identity, affect, and peer relations in young athletes with physical disabilities. Disabil Health J.

[18] Behle A, Pinquart M (2015). Perceived attainment of developmental tasks in adolescents with and without physical disabilities. J Dev Phys Disabil.

[19] Holmbeck G, Johnson S, Wills K, McKernon W, Rose B, Erklin S, Kemper T (2002). Observed and perceived parental overprotection in relation to psychosocial adjustment in preadolescents with a physical disability: The mediational role of behavioral autonomy. Clin Adolesc Psychol.

[20] Sanders K (2006). Overprotection and lowered expectations of persons with disabilities: The unforeseen consequences. Work.

[21] Larson R, Angus R (2011). Adolescents’ development of skills for agency in youth programs: Learning to think strategically. Child Dev.

[22] Kingsnorth S, Healy H, MacArthur C (2007). Preparing for adulthood: A systematic review of life skill programs for youth with physical disabilities. J Adolesc Health.

[23] Griffin M (1995). You can’t get there from here: Situated learning, transfer and map skills. Contemp Educ Psychol.

[24] King G, McPherson A, Kingsnorth S, Stewart D, Glencross-Eimantas T, Jones-Galley K, Gorter J (2015). Residential immersive life skills programs for youth with disabilities: Service providers’ perceptions of change processes. Disabil Rehabil.

[25] Kolb D, Boyatzis R, Mainemelis C (2001). Experiential learning theory: Previous research and new directions. Perspect Thinking Learn Cogn Styles.

[26] Standal Ø, Jespersen E (2008). Peers as resources for learning: A situated learning approach to adapted physical activity in rehabilitation. Adapt Phys Activ Q.

[27] McPherson A, Rudzik A, Kingsnorth S, King G, Gorter J, Morrison A (2016). “Ready to take on the world”: Experiences and understandings of independence after attending residential immersive life skills programs for youth with physical disabilities. Dev Neurorehabil.

[28] King G, McPherson A, Kingsnorth S, Stewart DG-ET, Gorter J, Jones-Galley K, Isihi A (2015). Residential immersive life skills programs for youth with disabilities: Service providers’ perceptions of experiential benefits and key program features. Disabil Rehabil.

[29] King G, Kingsnorth S, McPherson A, Jones-Galley K, Pinto M, Fellin M, Savage D. Residential immersive life skills programs for youth with physical disabilities: A pilot study of program opportunities, intervention strategies, and youth experiences. Research in Developmental Disabilities. 2016;55:242–55. 10.1016/j.ridd.2016.04.014 27153504

[30] Evans J, McDougall J, Baldwin P (2006). An evaluation of the “youth en route” program. Phys Occup Ther Pediatr.

[31] O’Cathain A, Murphy E, Nicholl J (2008). The quality of mixed methods studies in health services research. J Health Serv Res Policy.

[32] Fife-Schaw C, Breakwell G, Smith J, Wright D (2012). Quasi-experimental designs. Research methods in psychology.

[33] Wehmeyer M (1995). The Arc’s self-determination scale: Procedural guidelines.

[34] Wehmeyer M, Kelchner K (1995). Measuring the autonomy of adolescents and adults with mental retardation: A self-report form of the autonomous functioning checklist. Career Dev Except Individ.

[35] Scholz U, Dona B, Sud S, Schwarzer R (2002). Is general self-efficacy a universal construct? Psychometric findings from 25 countries. Eur J Psychol Assess.

[36] King G, Rigby P, Avery L (2015). Revised measure of environmental qualities of activity settings (MEQAS) for youth leisure and life skills activity settings. Disabil Rehabil.

[37] McDougall J, King G, Malloy-Miller T, Gritzan J, Tucker M, Evans J (1999). A checklist to determine the methods of intervention used in school-based therapy: Development and pilot testing. Phys Occup Ther Pediatr.

[38] King G, Batorowicz B, Rigby P, McMain-Klein M, Thompson L, Pinto M (2014). Development of a measure to assess youth self-reported experiences of activity settings (SEAS). Int J Disabil Dev Educ.

[39] Cohen J (1988). Statistical power analysis for the behavioral science.

[40] Lincoln Y, Guba E (1986). Naturalistic enquiry.

[41] Kingsnorth S, Rudzik A, King G, McPherson A (2016). Residential immersive life skills programs for youth with disabilities: A pilot study of youth and parent trajectories of personal growth Manuscript in preparation.

[42] Rosnow R, Rosenthal R (1996). Computing contrasts, effect sizes, and counternulls on other people’s published data: General procedures for research consumers. Pyschol Methods.

[43] Dierckx B, Gastmans C, Bryon E, Denier Y (2012). Quagol: A guide for qualitative analysis. Int J Nurs Stud.

[44] Avis M, Holloway I (2005). Is there an epistemology for qualitative research?. Qualitative research.

[45] Shenton A (2004). Strategies for ensuring trustworthiness in qualitative research projects. Educ Inf.

[46] Cresswell J (1998). Qualitative inquiry and research design: Choosing among the five traditions.

[47] Fade S, Swift J (2011). Qualitative research in nutrition and dietetics: Data analysis issues. J Hum Nutr Diet.

[48] Rolfe G (2006). Validity, trustworthiness and rigour: Quality and the idea of qualitative research. J Adv Nurs.

[49] Johnson R, Onwuegbuzie A, Turner L (2007). Toward a definition of mixed methods research. J Mixed Methods Res.

[50] Wisdom J, Cavaleri M, Onwuegbuzie A, Green C (2012). Methodological reporting in qualitative, quantitative, and mixed methods health services research articles. Health Serv Res.

[51] Canadian Institutes of Health Research. Guide to knowledge translation planning at CIHR: Integrated and end-of-grant approaches, 2012: Ottawa.

[52] Barwick M (2011). Knowledge translation planning template- r.

